# Organic materials repurposing, a data set for theoretical predictions of new applications for existing compounds

**DOI:** 10.1038/s41597-022-01142-7

**Published:** 2022-02-14

**Authors:** Ömer H. Omar, Tahereh Nematiaram, Alessandro Troisi, Daniele Padula

**Affiliations:** 1 University of Liverpool, Department of Chemistry, Liverpool, L69 7ZD UK; 2 Università di Siena, Dipartimento di Biotecnologie, Chimica e Farmacia, Siena, 53100 Italy

**Keywords:** Electronic materials, Density functional theory

## Abstract

We present a data set of 48182 organic semiconductors, constituted of molecules that were prepared with a documented synthetic pathway and are stable in solid state. We based our search on the Cambridge Structural Database, from which we selected semiconductors with a computational funnel procedure. For each entry we provide a set of electronic properties relevant for organic materials research, and the electronic wavefunction for further calculations and/or analyses. This data set has low bias because it was not built from a set of materials designed for organic electronics, and thus it provides an excellent starting point in the search of new applications for known materials, with a great potential for novel physical insight. The data set contains molecules used as benchmarks in many fields of organic materials research, allowing to test the reliability of computational screenings for the desired application, “rediscovering” well-known molecules. This is demonstrated by a series of different applications in the field of organic materials, confirming the potential for the repurposing of known organic molecules.

## Abstract (summary)

**Table:** 

Measurement(s)	excited state energy
Technology Type(s)	quantum chemistry computational method

Machine-accessible metadata file describing the reported data: 10.6084/m9.figshare.17076254

## Background & Summary

High Throughput Virtual Screenings (HTVSs)^1,2^ have recently been exploited to a great extent to identify promising materials in the domain of organic electronics. This powerful technique has often been used in combination with domain knowledge of the problem, carrying out screenings of modifications of known motifs or architectures known to work for a specific problem *e.g*. functionalisation for dye-sensitized solar cells^3^, donor-acceptor motifs for thermally activated delayed fluorescence (TADF)^4^, singlet fission (SF)^5^, and for general photovoltaic architectures^6^. This strategy translates in computational terms the process of experimental discovery exploiting chemical intuition^7,8^, and allows the reduction of the chemical space to explore^9^. However, the findings are bound to fall within the domain of what is already known and prevent the discovery of new motifs and design rules. Studies based on exploiting domain knowledge like biradical character for SF^10,11^ or donor-acceptor motifs for TADF^12,13^ will not find new design rules. Generative models also tend to find motifs similar to those already known^14^. Additionally, the identified candidates may not be easy to synthesise in the laboratory or be stable enough to be characterised, despite recent progresses in introducing measures of synthetic accessibility in HTVSs^15^.

In this study, we aim at providing a starting point for computational searches overcoming the mentioned limitations by presenting a data set of 48182 organic semiconductors (OSCs) constituted of molecules that were prepared with a documented synthetic pathway, and are stable in solid-state, enabling their crystallographic characterisation. The data set is therefore an excellent starting point to identify OSCs for various applications that can guide experimental research. We based our search on the Cambridge Structural Database (CSD)^16^, from which we selected OSCs with a computational strategy described in the following sections. The CSD dates back to the 60–70s, and contains crystal structure data for $$ > 1M$$ samples prepared for various purposes. Excluding polymorphs^17–19^ or samples measured in different experimental conditions^20^, the vast majority of molecules in the data set has characterisation data available. As it was not built with organic materials applications in mind though, of course, it does contain entries related to this field, any data set derived from the CSD^21^ is, therefore, unbiased with respect to the application, though some bias is present due to choices of research groups in the study of a certain molecule or experimental contraints with respect to the ability to crystallise the sample and characterise it. This low bias provides a great potential for novel physical insight: setting different criteria for the ideal candidates based on experimental benchmarks, the more stringent ones (*i.e*. more rare) can be used to translate results into design principles. Additionally, the fact that it contains molecules used for benchmarks in many fields of organic materials research allows testing the reliability of computational screenings for the desired application, “rediscovering” well known molecules.

Studies of OSCs for technological applications exploit the analyses of various electronic properties, ranging from frontier orbital energies to excited state energies and oscillator strengths. For instance, early searches of materials for organic photovoltaics exploited HOMO and LUMO energies^22–25^, high performance non-fullerene acceptors are known to possess a low LUMO-LUMO + 1 gap^26,27^, luminescent materials for new generation organic light emitting diodes (OLEDs) based on TADF^28,29^ as well as singlet fission candidates^30^ have been identified by calculating the S_1_-T_1_ gap ($\Delta {E}_{ST}$), and high mobility semiconductors were discovered by looking at electronic couplings, reorganisation energies and electron-phonon couplings^31–33^. Providing wavefunctions and basic excited state properties for the first few states will enable other researchers to carry out systematic investigations for applications that, to the best of our knowledge, are yet to be explored through computational screenings, such as aggregation induced emitters (AIEgens)^34^, but also for extremely innovative applications based on higher excited states, for which chemical intuition is still limited, *e.g*. designing anti-Kasha fluorophores^35^, even displaying delayed fluorescence^36^.

The data set presented in this work thus contains a collection of simulated spectroscopic properties on the X-ray geometries of existing organic molecules, showing a simulated HOMO-LUMO gap (${E}_{gap}$) falling below 4 eV, which we therefore define as organic semiconductors, and can be searched for relevant properties in various technological applications. Some data sets offer interesting properties for OSCs relevant for specific applications, *e.g.* the HOPV for organic photovoltaics^37^, but they are limited to boundaries within the chemical space, *i.e*. they exploit domain knowledge about what is known to work. Other data sets offer spectroscopic properties of molecules, such as *e.g* the QM8^38^ or the OE62^21^ data sets, but the former is limited in the number and type of heavy atoms and excited states considered, while the latter provides spectroscopic data only for a small fraction of the data set ($\approx 5K$ entries). The data set we present in this work is thus aimed at complementing the currently available ones in these aspects, which we describe in more detail in the following sections.

## Methods

The data set of OSCs we present here has been built starting from the python application programming interface provided within the CSD distribution. To identify OSCs, we started by removing polymeric molecules, disordered solids, and co–crystals from the entries containing X-ray structures. We further reduced the structures to be retained by:

1. including only the most commonly used elements in typical OSCs in the selection of molecules (H, B, C, N, O, F, Si, P, S, Cl, As, Se, Br, I);
2. removing entries with more than one molecule type in the unit cell;
3. removing duplicate entries

X-ray geometries include all heavy atoms, while hydrogen atoms are added and normalised (*i.e*. placed at a typical X-H distance using statistical surveys of neutron diffraction data) using the CSD library’s built-in functions exploiting such literature data^39,40^. Due to errors in the procedure, *e.g*. missing hydrogens in diborane moieties, the structurally erroneous entries are filtered out by comparing the heavy atom connectivity layer of InChI^41^ strings of the CSD entry and the extracted geometry, followed by comparison of the chemical formulae between the CSD entry and the extracted geometry. The data set is up to date with the 2020 version of the CSD, thus updates starting from the 2021 version are possible.

**Fig. 1:**
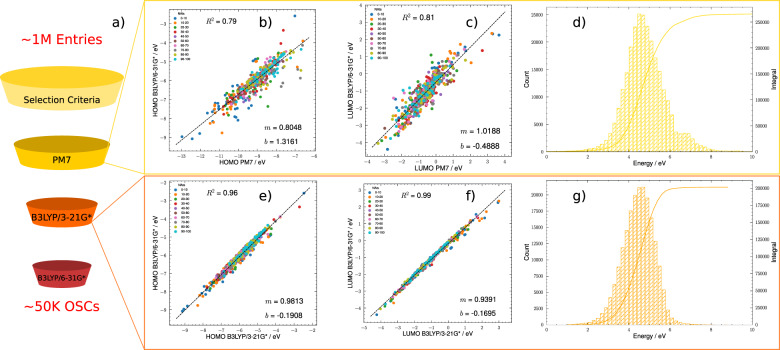
(**a**) Computational strategy used to identify OSCs starting from the CSD. (**b**) Calibration curve to estimate B3LYP/6-31 G* HOMO from PM7 HOMO. (**c**) Calibration curve to estimate B3LYP/6-31 G* LUMO from PM7 LUMO. (**d**) distribution of estimated B3LYP/6-31 G* HOMO-LUMO gap from PM7 energy levels. (**e**) Calibration curve to estimate B3LYP/6-31 G* HOMO from B3LYP/3-21 G* HOMO. (**f**) Calibration curve to estimate B3LYP/6-31 G* LUMO from B3LYP/3-21 G* LUMO. (**g**) distribution of estimated B3LYP/6-31 G* HOMO-LUMO gap from B3LYP/3-21 G* energy levels.

**Fig. 2:**
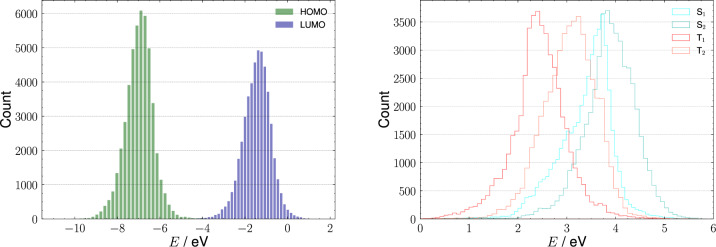
Distributions of energy levels computed on X-ray geometries for all entries in the database. Left: frontier molecular orbitals computed at the DFT/M06-2X/def2-SVP level. Right: singlet (S_1_, S_2_) and triplet (T_1_, T_2_) excited state energies computed at TD-DFT/M06-2X/def2-SVP level.

This procedure resulted in a reduction of the data set from $\approx 1M$ to $\approx 265K$ molecules. To identify OSCs we adopted a three-step computational funnel strategy in combination with a calibration procedure, aimed at estimating the HOMO-LUMO gap (${E}_{gap}$) with quantum mechanical (QM) methods of reasonable accuracy. First of all, we selected three methods of increasing accuracy for our computational funnel: PM7, B3LYP/3 – 21 G*, and B3LYP/6–31 G*. Second, we picked a subset of 550 molecules on which we performed single point calculations on the X-ray geometries provided within the CSD, obtaining orbital energies with all three methods. This allowed us to compute calibration curves to estimate the B3LYP/6-31 G* HOMO-LUMO gap (${\widetilde{E}}_{gap}$) from low accuracy ones (see panels b), c), e), and f) in Fig. 1), and the associated error distribution. With calibration curves available, we proceeded to compute HOMO-LUMO gaps (${\widetilde{E}}_{gap}$) for the entire data set of $\approx 265K$ molecules (panel d) in Fig. 1), estimating the gap that we would obtain if we ran a higher level calculation. Considering the distribution of errors of the calibration curve, at the PM7 level we retained any molecule showing ${\widetilde{E}}_{gap}\le 5.5$ eV as a potential OSC, reducing the data set from $\approx 265K$ to $\approx 200K$ molecules. On these molecules, we recomputed the gap at the B3LYP/3-21 G* level (panel g) in Fig. 1), considering any molecule showing ${\widetilde{E}}_{gap}\le 4$ eV as an OSC, resulting in the $\approx 50K$ molecules that constitute the data set presented here. 4 eV is a conventional upper limit for semiconductors^42^, and all the best performing molecules across various applications have a smaller gap. On these molecules, we computed excited states properties at TD-DFT/M06-2X/def2-SVP (see Fig. 2), releasing, as part of the data set, the converged ground state wavefunction, and the results for the first three singlet (S_1_-S_3_) and triplet (T_1_-T_3_) states. A calibration of the TD-DFT method for S_1_ and T_1_ excitation energies for $\approx 100$ data points with available experimental data is presented elsewhere^30^, and guarantees the reliability of the method (RMSE $\approx 0.05$ eV). All QM calculations were carried out with the Gaussian16 software^43^, and the data provided as part of this release were extracted from output and checkpoint files using the Multiwfn software^44^ and the CClib python library^45^.

**Fig. 3:**
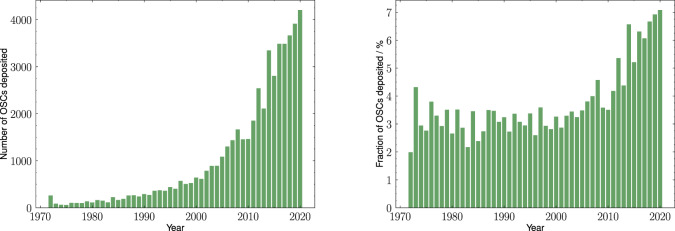
Time evolution of OSCs within the CSD. Left: total number of OSCs deposited each year. Right: fraction of OSCs deposited each year.

These calculations allow for interesting analyses regarding the time evolution of the CSD. For instance, since the deposition date of each entry is known, it is possible to follow how many OSCs were deposited over time, both in absolute and fractional terms. From these analyses (see Fig. 3) we see that, while the absolute number is naturally increasing over time, the fractional number of OSCs within the CSD is constant until ≈2010, and since then it has basically doubled, rising from ≈3–4% to ≈7%, which is in agreement with the evolution of research in the organic materials field.

## Data Records

The curated data set is available from DataCat, the University of Liverpool repository^46^:

1. data extracted from QM calculations are provided at the University of Liverpool repository^46^ in comma-separated values (.csv) format, which can be easily read through common programs or programming languages. A description of the provided data is given in Table 1;

**Table 1:** Description of metadata and electronic properties gathered in the database.

Column name	Unit	Method	Description
ID	—	—	unique CSD identifier
doi	—	—	doi of the experimental paper characterising the X-ray structure
formula	—	—	chemical formula
NAts	—	—	number of heavy atoms
SMILES	—	—	the SMILES string^67–69^
HOMO	eV	TD-DFT/M06-2X/def2-SVP	computed HOMO energy on the X-ray geometry
LUMO	eV	TD-DFT/M06-2X/def2-SVP	computed LUMO energy on the X-ray geometry
E(S1)	eV	TD-DFT/M06-2X/def2-SVP	computed S1 energy on the X-ray geometry
f(S1)	—	TD-DFT/M06-2X/def2-SVP	computed S1 oscillator strength on the X-ray geometry
E(S2)	eV	TD-DFT/M06-2X/def2-SVP	computed S2 energy on the X-ray geometry
f(S2)	—	TD-DFT/M06-2X/def2-SVP	computed S2 oscillator strength on the X-ray geometry
E(S3)	eV	TD-DFT/M06-2X/def2-SVP	computed S3 energy on the X-ray geometry
f(S3)	—	TD-DFT/M06-2X/def2-SVP	computed S3 oscillator strength on the X-ray geometry
E(T1)	eV	TD-DFT/M06-2X/def2-SVP	computed T1 energy on the X-ray geometry
E(T2)	eV	TD-DFT/M06-2X/def2-SVP	computed T2 energy on the X-ray geometry
E(T3)	eV	TD-DFT/M06-2X/def2-SVP	computed T3 energy on the X-ray geometry
2. the wavefunctions for each entry are provided in a set of 31 sequential archives at the University of Liverpool repository^46^ allowing for sequential or partial download. Geometries are also given to facilitate analyses. Data are made available in .wfn format;
3. Gaussian16 QM calculations output files are provided at the University of Liverpool repository^46^ to allow for additional wavefunction analyses, with the aim to characterise electronic states or transitions, as mentioned in the following sections.

Geometries and wavefunctions are provided in .wfn format, the AIM traditional format. We chose this format to provide interested users data for analyses or subsequent calculations that would be independent of the software we used. In fact, .wfn files can be generated or processed with a multitude of tools, among which the popular software Multiwfn^44^, the python library IOData^47^, ORCA^48,49^ and others^50,51^. Each .wfn file contains the molecular geometry, as well as occupied molecular orbitals expressed in the atomic basis and their energies. These data can be used for visualisation of *e.g*. geometries, occupied orbitals, but also to run QM calculations with an initial guess to obtain refined properties for applications of interest.

Gaussian16 output files are provided to allow for additional wavefunction analyses on electronic excitations, allowing interested users to avoid repeating calculations that we have already performed.

## Technical Validation

The key idea is that new applications of existing molecules can be discovered by searching for useful properties computed for a large data set, thanks to a robust calibration between predicted and experimentally validated data. Crucially, the data set should be totally unbiased and not related to the property of interest: this way, discoveries are truly unexpected and have a large applicative and commercial value. We proved this concept through a range of demonstrations in recent works, covering various applications areas. These demonstrations considered an outdated data set consisting of $\approx 40K$ OSCs. The data set presented here is up to date with the 2020 version of the CSD, thus containing entries that were not the objects of our previous studies; the same strategies can be used on the fraction of molecules not previously considered to discover more potential candidates, in line with our previous findings.

The key applications demonstrated in our previous works are the following:

1. we showed that it is possible to identify completely new molecules that undergo singlet fission (a property of relevance for solar cells) by calibrating a computational method to yield accurate energies of singlet and triplet excited states and found molecules with the ideal energy level alignment^30^. The method rediscovered known molecules for singlet fission (true positives), and identified several different families of known compounds with this desirable property;
2. we proposed a related screening protocol to identify molecules undergoing TADF^28^, a relevant property in the area of display technologies. The protocol indicated without any adjustable parameter that $0.3 \%$ of the $\approx 40K$ molecules considered may undergo TADF. About half of them were known TADF emitters, providing great confidence in the quality of the prediction. The other half of the hits were totally unknown to the field, illustrating in parallel how this approach can lead to completely novel design rules;
3. we showed that a similar approach can be used to identify novel electron acceptors to be used in organic solar cells to replace expensive and inefficient fullerene derivatives^52^. Also in this case, about half of the “discovered” molecules were known, the other half being totally novel ones. This work showed that database searching is only the first step and it is possible to modify lead compounds to have other desirable properties, like solubility;
4. we showed that we can screen for luminescent crystals displaying superradiance or near IR emission^53^, properties of interest in the areas of light-emitting diodes, organic lasers, and biological imaging. A common theme of all applications particularly well exemplified by this one is the ability of large screenings to identify plausible optima for any properties; in this case, what is the maximum red shift that can be observed when a particular molecule is studied in its crystal.

**Fig. 4:**
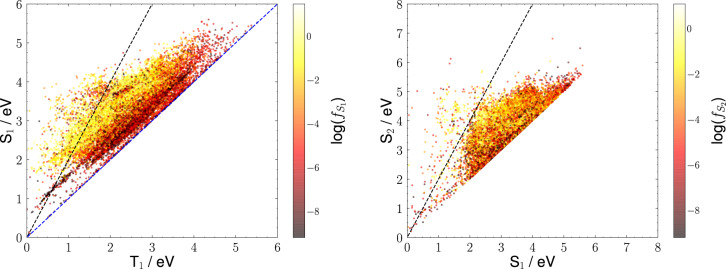
Relationships between relevant excited states energies to identify promising materials within the database. Left: T_1_ *vs* S_1_ energies. Potential singlet fission materials fall in proximity of the dashed black line, representing S_1_ = 2 T_1_. Potential TADF materials fall in proximity of the dashed blue line, representing S_1_ = T_1_. Colours encode the S_1_ oscillator strength (${f}_{{S}_{1}}$) through a logarithmic scale, assuming bright states are desirable. Right: S_1_ *vs* S_2_ energies. Potential anti-Kasha materials fall in proximity of the dashed black line, representing S_2_ = 2 S_1_. Colours encode the S_2_ oscillator strength (${f}_{{S}_{2}}$) through a logarithmic scale, assuming bright states are desirable.

The basis of similar studies can be laid by analysing properties provided in this database similarly to what is shown in Fig. 4. In the left panel, we report T_1_ *vs* S_1_ energies. Potential singlet fission materials fall to the left of the dashed black line, representing the main singlet fission criterion, *i.e*. S_1_ = 2 T_1_. Similarly, potential TADF materials fall in the proximity of the dashed blue line, representing the main TADF criterion, *i.e*. S_1_ = T_1_. Colours encode the S_1_ oscillator strength (${f}_{{S}_{1}}$) through a logarithmic scale, since one would be interested in materials able to absorb (singlet fission) or emit (TADF) light with a good performance. These types of analyses led us to the work shortly described in points 1 and 2, where we have “rediscovered” well known singlet fission and TADF materials, proving that the starting point, *i.e*. a reduced version of the data set presented here, is reliable. The same, however, can be done for other properties yet to be studied: for instance, in the right panel of Fig. 4, we report S_1_ *vs* S_2_ energies, useful to identify potential anti-Kasha materials, falling in the proximity of the dashed black line, representing S_2_ = 2 S_1_. This is a reasonable criterion according to domain knowledge regarding the role of kinetics in anti-Kasha photoreactions^54,55^. In this case, colours encode the S_2_ oscillator strength (${f}_{{S}_{2}}$) through a logarithmic scale, since in anti-Kasha materials the fluorescence is expected from a higher excited state.

## Usage Notes

Above, we have listed some applications deriving from the data presented here. In general, the starting point for each of those applications consisted of a calibration of the computational method used to carry out further analyses with available experimental data. Thanks to the fact that we provide the ground state wavefunction for each of our entries, not only will these calibrations be faster because we provide an initial guess for QM calculations, but also many more analyses are accessible. For instance, electronic states or transitions can be thoroughly characterised with packages such as Multiwfn^44^ or TheoDORE^56^, which can provide detailed information regarding the nature of an electronic transition (*e.g*. Charge Transfer metrics^57,58^, ghost states^59^, electronic density difference^60^, exciton delocalisation^61,62^ *etc*). Additionally, this data set can form the basis for training sets to Machine Learning models aiming at reproducing the electronic density of molecules^63,64^, based on experimental X-ray geometries. The availability of CSD identifiers enables the expansion of analyses to molecules in their crystals^32^, which is fundamental for technological applications of organic semiconductors. Finally, the synthetic approaches that make molecules within the CSD accessible can be easily tracked down thanks to references provided within the data set. This allows not only for a prompt source of synthetic routes to be exploited in case of experimental validation of the results, but is also useful in combination with retrosynthetic planning strategies^65,66^.

## Data Availability

Scripts to obtain plots starting from the database are available at the University of Liverpool repository^46^.

## References

[1] Pyzer-Knapp EO, Suh C, Gómez-Bombarelli R, Aguilera-Iparraguirre J, Aspuru-Guzik A (2015). What is high-throughput virtual screening? a perspective from organic materials discovery. Annu. Rev. Mater. Res..

[2] Omar ÖH, del Cueto M, Nematiaram T, Troisi A (2021). High-throughput virtual screening for organic electronics: A comparative study of alternative strategies. J. Mater. Chem. C.

[3] Ørnsø KB, Pedersen CS, Garcia-Lastra JM, Thygesen KS (2014). Optimizing porphyrins for dye sensitized solar cells using large-scale ab initio calculations. Phys. Chem. Chem. Phys..

[4] Shu Y, Levine BG (2015). Simulated evolution of fluorophores for light emitting diodes. J. Chem. Phys..

[5] Blaskovits JT, Fumanal M, Vela S, Corminboeuf C (2020). Designing singlet fission candidates from donor–acceptor copolymers. Chemistry of Materials.

[6] Jørgensen PB (2018). Machine learning-based screening of complex molecules for polymer solar cells. J. Chem. Phys..

[7] Padula D, Troisi A (2019). Concurrent optimisation of organic donor-acceptor pairs through machine learning. Adv. Energy Mater..

[8] Padula D, Simpson JD, Troisi A (2019). Combining electronic and structural features in machine learning models to predict organic solar cells properties. Mater. Horiz..

[9] von Lilienfeld OA (2018). Quantum machine learning in chemical compound space. Angew. Chem. Int. Ed..

[10] Minami T, Nakano M (2011). Diradical character view of singlet fission. J. Phys. Chem. Lett..

[11] Omar ÖH, Padula D, Troisi A (2020). Elucidating the relationship between multiradical character and predicted singlet fission activity. ChemPhotoChem.

[12] Tanaka H, Shizu K, Nakanotani H, Adachi C (2013). Twisted intramolecular charge transfer state for long-wavelength thermally activated delayed fluorescence. Chem. Mater..

[13] Zhang Y (2016). Supramolecular structure-dependent thermally-activated delayed fluorescence (TADF) properties of organic polymorphs. J. Phys. Chem. C.

[14] Sanchez-Lengeling B, Aspuru-Guzik A (2018). Inverse molecular design using machine learning:Generative models for matter engineering. Science.

[15] Wen, Y., Fu, L., Li, G., Ma, J. & Ma, H. Accelerated Discovery of Potential Organic Dyes for Dye-Sensitized Solar Cells by Interpretable Machine Learning Models and Virtual Screening. *Solar RRL* **4**, 10.1002/solr.202000110 (2020).

[16] Groom CR, Bruno IJ, Lightfoot MP, Ward SC (2016). The cambridge structural database. Acta Cryst..

[17] Landi A, Troisi A, Peluso A (2019). Explaining different experimental hole mobilities: influence of polymorphism on dynamic disorder in pentacene. J. Mater. Chem. C.

[18] Mattheus CC, de Wijs GA, de Groot RA, Palstra TTM (2003). Modeling the polymorphism of pentacene. J. Am. Chem. Soc..

[19] Mattheus CC (2001). Polymorphism in Pentacene. Acta Cryst. Sect. C.

[20] Siegrist T (2007). A polymorph lost and found: The high-temperature crystal structure of pentacene. Adv. Mater..

[21] Stuke, A. *et al*. Atomic structures and orbital energies of 61,489 crystal-forming organic molecules. *Scientific Data* **7**, 10.1038/s41597-020-0385-y (2020). 10.1038/s41597-020-0385-y PMC7029047 32071311

[22] Pyzer-Knapp EO, Li K, Aspuru-Guzik A (2015). Learning from the harvard clean energy project: The use of neural networks to accelerate materials discovery. Adv. Funct. Mater..

[23] Hachmann J (2014). Lead candidates for high-performance organic photovoltaics from high-throughput quantum chemistry – the harvard clean energy project. Energy Environ. Sci..

[24] Hachmann J (2011). The harvard clean energy project: Large-scale computational screening and design of organic photovoltaics on the world community grid. J. Phys. Chem. Lett..

[25] Kanal IY, Owens SG, Bechtel JS, Hutchison GR (2013). Efficient computational screening of organic polymer photovoltaics. J. Phys. Chem. Lett..

[26] Kuzmich A, Padula D, Ma H, Troisi A (2017). Trends in the electronic and geometric structure of non-fullerene based acceptors for organic solar cells. Energy Environ. Sci..

[27] Liu, T. & Troisi, A. What makes fullerene acceptors special as electron acceptors in organic solar cells and how to replace them. *Adv. Mater.* **25**(7), 1038–1041, 10.1002/adma.201203486 (Wiley, nov 2012). 10.1002/adma.201203486 23192958

[28] Zhao K, Ömer HO, Nematiaram T, Padula D, Troisi A (2021). Novel thermally activated delayed fluorescence materials by high-throughput virtual screening: going beyond donor–acceptor design. J. Mater. Chem. C.

[29] Gómez-Bombarelli R (2016). Design of efficient molecular organic light-emitting diodes by a high-throughput virtual screening and experimental approach. Nat. Mater..

[30] Padula D, Omar ÖH, Nematiaram T, Troisi A (2019). Singlet fission molecules among known compounds: Finding few needles in a haystack. Energy Environ. Sci..

[31] Landi A, Peluso A, Troisi A (2021). Quantitative prediction of the electro-mechanical response in organic crystals. Adv. Mater..

[32] Nematiaram T, Padula D, Landi A, Troisi A (2020). On the largest possible mobility of molecular semiconductors and how to achieve it. Adv. Funct. Mater..

[33] Schober C, Reuter K, Oberhofer H (2016). Virtual screening for high carrier mobility in organic semiconductors. J. Phys. Chem. Lett..

[34] Hong Y, Lam JWY, Tang BZ (2011). Aggregation-induced emission. Chem. Soc. Rev..

[35] Shi, L. *et al*. De novo strategy with engineering anti-kasha/kasha fluorophores enables reliable ratiometric quantification of biomolecules. *Nat. Commun*. **11**, 10.1038/s41467-020-14615-3 (2020). 10.1038/s41467-020-14615-3 PMC7005775 32034152

[36] Jhun BH, Jeong DY, Nah S, Park SY, You Y (2021). Novel anti-kasha fluorophores exhibiting dual emission with thermally activated delayed fluorescence through detouring triplet manifolds. J. Mater. Chem. C.

[37] Lopez, S. A. *et al*. The harvard organic photovoltaic dataset. *Sci. Data* **3**, 10.1038/sdata.2016.86 (2016). 10.1038/sdata.2016.86 PMC5037972 27676312

[38] Ramakrishnan R, Hartmann M, Tapavicza E, von Lilienfeld OA (2015). Electronic spectra from TDDFT and machine learning in chemical space. J. Chem. Phys..

[39] Allen FH (1987). Tables of bond lengths determined by x-ray and neutron diffraction. part 1. bond lengths in organic compounds. J. Chem. Soc. Perkin Trans..

[40] Allen FH, Bruno IJ (2010). Bond lengths in organic and metal-organic compounds revisited:x—h bond lengths from neutron diffraction data. Acta. Crystallogr. B. Struct. Sci. Cryst. Eng. Mater..

[41] Heller, S., McNaught, A., Stein, S., Tchekhovskoi, D. & Pletnev, I. InChI - the worldwide chemical structure identifier standard. *J. Cheminformatics* **5**, 10.1186/1758-2946-5-7 (2013). 10.1186/1758-2946-5-7 PMC3599061 23343401

[42] Costa JC, Taveira RJ, Lima CF, Mendes A, Santos LM (2016). Optical band gaps of organic semiconductor materials. Optical Materials.

[43] Frisch, M. J. *et al*. *Gaussian 16 Revision C.01 (2016)*. Gaussian Inc. Wallingford CT.

[44] Lu T, Chen F (2011). Multiwfn: A multifunctional wavefunction analyzer. J. Comput. Chem..

[45] O'boyle NM, Tenderholt AL, Langner KM (2008). cclib: A library for package-independent computational chemistry algorithms. J. Comput. Chem..

[46] Omar ÖH, Nematiaram T, Troisi A, Padula D (2021). DataCat, University of Liverpool.

[47] Verstraelen T (2021). IOData: A python library for reading, writing, and converting computational chemistry file formats and generating input files. J. Comput. Chem..

[48] Neese F (2011). The ORCA program system. WIREs Comput. Mol. Sci..

[49] Neese, F. Software update: the ORCA program system, version 4.0. *WIREs Comput. Mol. Sci*. **8**, 10.1002/wcms.1327 (2017).

[50] Hermann G (2016). ORBKIT: A modular python toolbox for cross-platform postprocessing of quantum chemical wavefunction data. J. Comput. Chem..

[51] de-la Roza AO, Johnson ER, Luaña V (2014). Critic2: A program for real-space analysis of quantum chemical interactions in solids. Comput. Phys. Commun..

[52] Zhao Z-W, Omar ÖH, Padula D, Geng Y, Troisi A (2021). Computational identification of novel families of nonfullerene acceptors by modification of known compounds. J. Phys. Chem. Lett..

[53] Nematiaram T, Padula D, Troisi A (2021). Bright frenkel excitons in molecular crystals: A survey. Chem. Mater..

[54] Demchenko AP, Tomin VI, Chou P-T (2017). Breaking the kasha rule for more efficient photochemistry. Chem. Rev..

[55] Tomin VI, Dubrovkin JM (2017). Kinetics of anti-kasha photoreactions. direct excitation of a higher excited state. ChemistrySelect.

[56] Plasser F (2020). TheoDORE: A toolbox for a detailed and automated analysis of electronic excited state computations. J. Chem. Phys..

[57] Guido CA, Cortona P, Mennucci B, Adamo C (2013). On the metric of charge transfer molecular excitations: A simple chemical descriptor. J. Chem. Theory Comput..

[58] Padula D, Di Bari L, Pescitelli G (2016). The “case of two compounds with similar configuration but nearly mirror image CD spectra” refuted. reassignment of the absolute configuration of n-formyl-3′,4′-dihydrospiro[indan-1,2′(1′ h)-pyridine]. J. Org. Chem..

[59] Campetella M (2017). Charge transfer excitations in TDDFT: A ghost-hunter index. J. Comput. Chem..

[60] Campetella M, Perfetto A, Ciofini I (2019). Quantifying partial hole-particle distance at the excited state: A revised version of the DCT index. Chem. Phys. Lett..

[61] Padula D, Jurinovich S, Di Bari L, Mennucci B (2016). Simulation of electronic circular dichroism of nucleic acids: From the structure to the spectrum. Chem. Eur. J..

[62] Mewes SA, Mewes J-M, Dreuw A, Plasser F (2016). Excitons in poly(para phenylene vinylene): a quantum-chemical perspective based on high-level ab initio calculations. Phys. Chem. Chem. Phys..

[63] Schütt, K. T., Gastegger, M., Tkatchenko, A., Müller, K.-R. & Maurer, R. J. Unifying machine learning and quantum chemistry with a deep neural network for molecular wavefunctions. *Nat. Commun*. **10**, 10.1038/s41467-019-12875-2 (2019). 10.1038/s41467-019-12875-2 PMC6858523 31729373

[64] Gastegger M, McSloy A, Luya M, Schütt KT, Maurer RJ (2020). A deep neural network for molecular wave functions in quasi-atomic minimal basis representation. J. Chem. Phys..

[65] Segler MHS, Preuss M, Waller MP (2018). Planning chemical syntheses with deep neural networks and symbolic AI. Nature.

[66] Genheden, S. *et al*. AiZynthFinder: a fast, robust and flexible open-source software for retrosynthetic planning. *J. Cheminformatics* **12**, 10.1186/s13321-020-00472-1 (2020). 10.1186/s13321-020-00472-1 PMC7672904 33292482

[67] Weininger D (1988). SMILES, a chemical language and information system. 1. introduction to methodology and encoding rules. J. Chem. Inf. Comput. Sci.

[68] Weininger D, Weininger A, Weininger JL (1989). SMILES. 2. algorithm for generation of unique SMILES notation. J. Chem. Inf. Comput. Sci.

[69] Weininger D (1990). SMILES. 3. DEPICT. graphical depiction of chemical structures. J. Chem. Inf. Comput. Sci.

